# Proceedings

**Published:** 1886-12

**Authors:** E. G. Betty


### THE DENTAL REGISTER.

VOL. XL.] DECEMBER, 1886. [No. 12.

### PROCEEDINGS OF SOCIETIES.

### Discussions at the Second Annual Meeting of the Ohio State Dental Society, Reorganized, at Toledo, Ohio, October 26th, 27th, and 28th, 1886.

REPORTED BY E. G. BETTY, D.D.S.

The second annual meeting of this Society was called to order in the City Council Chamber by President Rehwinkel at 10:30 A. M., the entire morning session being devoted to the transaction of routine business and the election of new members, the minutes of which will be found elsewhere in the REGISTER.

AFTERNOON SESSION.

*Dr. C. R. Butler* read the following paper upon the first subject upon the programme:

WANT OF ROOM FOR THE ERUPTION OF THE PERMANENT TEETH, ESPECIALLY THE THIRD MOLARS.

Rarely do we see any marked irregularity in the arrangement of the temporary teeth. But it is not so with the permanent. A fairly arranged set of teeth at the age of eighteen or twenty years, among Americans, is not the rule, but rather the exception.

The first molars of the permanent set are generally erupted when the child is six years old, with crowns rather more bulky than the molars that appear later. And these serve a very important function. Their roots being completely formed before the temporary molars are shed, they sustain the force of mastication between childhood and youth, thus giving the normal stimulus to the salivary glands, that the food may be thoroughly insalivated before deglutition.

They also prevent undue pressure upon the but partially developed teeth of replacement. The jaws having sufficiently elongated, these teeth take position just back of the twenty temporary ones.

Within the next six years, the jaws elongate backwards, to make room for the second, and for the third molars. And, of the later, I desire now to speak more particularly of the lower ones. Being situated in the angle of the inferior maxillary bones, much discomfort, and often serious trouble is experienced, for lack of room for their eruption.

The general structure and arrangement of the full complement of thirty-two teeth, gives a fair index to the anatomical and physiological make-up of the individual.

The shortening and narrowing of the lower portion of the face, and the want of room in the maxillary bones, in the American race of to-day, are apparent to even the casual observer. And the multitude of ailments that confront the general practitioner, and the specialist as well, are found to be more and more of a nervous character.

To ascertain the appropriate remedies, and the best methods of applying them, for the alleviation of disease, and the correction of deformity, is the prime object of our present coming together.

Doubtless all have realized the truth of the text, "Behold how great a matter a little fire kindleth;" and a tooth is often the cause of serious trouble; not only local, but general, through nerve irritation.

The typal number being still retained, and the size of the teeth not decreased in proportion to the restricted territory prepared for their reception, a writer has claimed that we have, in these facts, the explanation of many cases of irregularity and decay of the teeth. The trouble often lies in the inheritance of incongruous jaws and teeth from the parents. The father may have large teeth in a corresponding jaw, and the mother, small ones in a small jaw; and while these both may have perfect dentures, the inheritance may be most unfortunate for the child. The teeth being inherited from the father, and developed in a small jaw copied from the mother, the sad results are often seen in the mouths of children that come under our care.

In consequence of the limited space between the second molar and the base of the outer, or vertical portion of the ramus, the erupting third molar is forced against the lateral ends of a fixed circle. And the non-appearance of these teeth till the age of eighteen or twenty, or even when they present earlier or later, the inferior maxillary bone having become well solidified, and the preceding teeth being more or less crowded, serious inflammation is caused, demanding surgical or other relief.

The text-books and periodicals of dental surgery give but very little, if any suggestive treatment for such cases, except the removal of the second molar, to make room for the imprisoned crown, or treatment that would, as a rule, show a want of judgment and skill equal to that of the general surgeon who would advocate amputation in fracture of the fore-arm, before trying to save the injured member by conservative treatment.

To sacrifice a tooth of the most important class, to make room for one on the extreme end of the jaw, and questionable make-up, with only a hope of useful occlusion, seems unwise except in extreme cases. If the second molar is badly decayed, its removal would be plainly indicated.

The dento-maxillary nerves, being distributed from the gesserian ganglion, it is easy to see how reflex nerve irritation is caused by one of these offending teeth.

Having had quite a number of these troublesome cases present for relief within the past year, I thought it might not be altogether uninteresting to make them the basis of a brief paper, hoping that it might be the means of giving me some valuable hints. Situated as they are, it is not strange that we should ofton find aggravated throat troubles, deafness, stiffness of the muscles of the neck and face, with facial neuralgia, local paralysis, prostrating pain, culminating in severe abscess, with other local and systemic disturbances, caused by one or more of these impacted teeth.

Young women often suffer severely from these teeth, especially at the approach of the menstrual periods. I could mention some special cases in which the distress was great. Some had suffered from nervous troubles that did not yield to general treatment. One had, for some time, complained of a dull pain in the back of head and neck, had often difficulty in swallowing, she became alarmed, had consulted noted specialists, and to use her own language, "I feel at times as though I should go crazy, this pain depresses me so."

The third molars, above and below, were removed, and in a short time she experienced general relief, with freedom in swallowing. In two other cases of similar trouble, relief was obtained by removing the inferior ones.

Often palliative treatment must be adopted for a time, depleting the gum, or clipping away the portion overlaying the tooth, will prove sufficient for present relief.

Extraction should not be resorted to while the parts are much inflamed, if it can be safely deferred.

With the great number of forceps that has been constructed, there is still great want of adaptation for the inferior teeth.

I have been on the lookout for years—had some made by Chevalier & Sherwood, and two pair by the celebrated Evrard, of London. From their small bulk they are well adapted for reaching the superior teeth.

Have removed some on the lower side, by cutting or excavating with the engine, rather than sacrifice a good second molar. I find the engine a most valuable adjunct in dividing roots for extracting. A crown may be quickly divided with a disk and sharp fissure bur.

General anasthesia is not favorable for such operations as the removal of the third molars.

*Dr. G. W. Keely.* The majority of these cases present themselves to us when the surrounding parts are in a state of severe inflammation, so that the pain upon the remaining teeth is very great, although, at the same time, general anaesthesia is contra indicated. My experience teaches me that the difficult eruption of the third molars is due to a crowded condition of the arch, and in such cases, it is my practice when the second molar is badly demoralized to remove it and thus allow the third molar to take its place, which it will do in nineteen out of every twenty cases. In one instance I just happen to think of, a young man, possessing a fine set of teeth, was confined to bed for six weeks, suffering great agony from the pain and swelling incident upon an impacted third molar. I always attack such cases with great timidity, on account of the danger of fracture, and because the pain of the operation is very great. Another dentist extracted the second molar for the gentleman, and afterwards blundered by taking out the third one also. In another case, that of a young girl, I did not deem it prudent to extract, so cut away a V shaped piece of the gum over the tooth half an inch in length, and subsequently removed another portion of soft tissue on the buccal side; this procedure afforded the relief sought. It is, however, very hard to decide just what to do in cases of this description, and we must be guided in a great measure by circumstances. If the second molar is badly decayed, it should always be removed in preference to the third molar. Here is a third molar with three well developed roots, and a fourth one smaller than the rest; there were two such teeth in the same mouth.

*Dr. H. H. Harrison.* I have here a specimen of an impacted third molar which was decayed badly, the pulp dead and the periosteum in a state of acute inflammation; the roots are badly turned, I removed the tooth by passing an elevator between it and the second molar and rotating it backwards.

*Dr. Siddall.* In Oberlin, there are between 1,200 and 1,500 students all the time, and at the age when they are erupting their third molars so that probably I see more impacted molars than others usually do. I use the physics forceps for their extraction, yet I am afraid of it, though I have not had an accident happen so far.

*Dr. C. H. Harroun.* In many cases it is necessary to extract the second molar to give relief. In some instances the third molar is covered with such a heavy portion of alveolar process that the only remedy is the extraction of the second molar. Again, sometimes the roots are so firmly imbedded that they mus tbe separated by means of the engine and suitable burs, so as to allow the introduction of a light elevator. Patients will readily submit to such an operation as this; as it is not near so painful as one would imagine. The upper third molars are more easily removed than the lower. The direction of the roots determines the facility or difficulty with which the teeth may be extracted.

*Dr. Butler.* I have here a forceps which is not very peculiar except that its bulk is very slight, thus permitting its introduction into the confined space incident upon the swollen condition of the parts. On the lower jaw the physics forceps will apply very nicely in many cases; but in the upper jaw there is great danger of fracture. Dr. Harroun is the first one to mention the use of the engine to separate the roots so that they can be be taken out; it is an application of sound common sense that I have now heard for the first time and have not seen in print.

*Dr. Harroun.* I did not think this method was at all unusual. I also resort to it to remove any obstreperous root; it can be done with very little pain and surprisingly easy.

*Dr. J. Taft.* I heartily approve of and wish to emphasize Dr. Harroun's remarks. His method just mentioned is new, and its efficiency is apparent upon the first glance. These third molars present to us a great variety of forms, shape, and position. Their roots almost always dip backward--very rarely forward, and it is this fact that facilitates their removal with the physic's forceps. The objection, however, to the use of this class of forceps, is the great liability of fracturing or abrading the posterior angle of the second molar. To avoid this contingency, an intervening substance should be used as a guard In some instances the roots may dip forward, or the crown may be covered with a heavy layer of bone, either one of which constitutes a great difficulty to be overcome in the successful extraction of the tooth. A trephine may be used to advantage in removing the covering of bone, the exposure of the root enabling the operator to grasp it with the forceps and extract it without the danger of an accident. Dr. Harroun's method perfectly meets such cases as these and should be resorted to rather than risk an extraction by the usual procedure.

It is well nigh impossible to use the chisel successfully in removing the superincumbent bone.

The third molars of the upper jaw are entirely different from those of the lower jaw. The form of this molar should be carefully compared with that of the other molars, in fact, all of the teeth, in order that we may gain some idea as to its probable shape. It sometimes happens that the third molar has three, four, or five roots, each one forming a sort of hook; such instances, however, are fortunately very rare.

The use of the engine with trephine and fissure burrs opens up a new feature.

*Dr. Jere. Robinson, Sr.* The roots of the lower third molar generally run almost directly backwards. For the extraction of these teeth I use a forceps, the beaks of which are nearly at a right angle with the handles, and by catching hold as near the second molar as possible, I lift upwards with slight rotation towards the septum, the roots will then naturally unlock. In the upper jaw, extract with a motion outward towards the cheek; in nineteen out of twenty cases you will thus have no trouble in performing a satisfactory operation.

*Dr. Keely.* In one case I had, not long ago, I must have caught the twentieth case my youthful friend Dr. Robinson mentions, as I tried in vain every means I could think of, and was obliged to give up in despair.

*Dr. Siddall.* I have here a physic's forceps that I have perfected as nearly as possible, though it may have to be changed somewhat yet; the beaks are concaved posteriorly.

*Dr. Lilly.* The only serious experience I have had was with a third molar tooth in my own mouth, it was badly decayed, and my son extracted it, the second molar also coming with it.

*Dr. J. Taft.* Impacted teeth are found in all positions in the mouth, the third molar most frequently being out of proper place, sometimes causing serious consequences, baffling the skill of the surgeon as well as that of the dentist. The whole list of lesions from irritation to inflammation, suppuration, and finally abscess may not infrequently occur. Sometimes an abscess of the first or second molar will extend to the third, involving it also, for the reason that its condition is favorable to it. All these cases demand careful examination and study. In looking over a collection of skulls, it is surprising to see the frequency of impaction of the third molar, occurring most often in the upper jaw.

*Dr. Potterf.* In my own mouth an impacted third molar has given me trouble for six or seven years, the tonsil of that side is often much swollen. I relieve the pain by making a crucial incision over the top of the tooth.

*Dr. J. Taft.* Are your third molars wholly covered by the gum?

*Dr. Potterf.* They are.

*Dr. J. Taft.* I have seen many cases where I believe the impaction was the cause of constantly recurring tonsilitis and inflammation of the throat; in some instances the throat trouble disappeared upon the extraction of the offending tooth.

*Dr. C. R. Taft.* The free use of the chisel is not so heroic as Dr. Harroun's method with the engine. I use the chisel freely, and with good stout blows from the mallet.

*Dr. J. R. Bell.* A domestic, subject to epileptic fits, occurring three or four times a week, consulted me, and upon examination, I found both inferior third molars impacted. The roots lay in a horizontal position, and the extraction was difficult though successfully performed. The fits gradually decreased in frequency, and now only occur once a month or every other month.

*Dr. H. A. Smith.* There would be considerably less trouble if the third molars could be erupted in earlier life, before the bone is thoroughly ossified. Their impaction is often the cause of obscure neuralgic pains, for which we can find no apparent cause, consequently we should be on the lookout for an impacted third molar. In extracting the upper third molar, the roots may be cut through with a disc—separated with the blade of a chisel and extracted. If we would take more time to study the details of the different methods of extracting these teeth, we could often save our patients a great deal more trouble and pain than if we relied exclusively on the forceps.

*Dr. J. Taft* now moved that Dr. Younger's pamphlet on the "Implantation of Teeth" be read.

*Dr. Metcalf* of Kalamazoo, Michigan, by request, read it.

*Dr. Harroun.* I have witnessed some of Dr. Younger's operations; he is fearless and expert as a surgeon, well educated, and it will not surprise me to see him make still more wonderful operations in this line.

*Dr. J. Taft.* The impression made on my mind, upon first hearing of this method, was that of incredulity. The statements in the first paper, and their reiteration in this, the second, leads me to the conclusion that he *has* attained the results he claims; if this is so, what will come next? Time alone will decide. I saw a case that goes a great ways towards verifying Dr. Younger's operations. It was one performed by Dr. Morrison, of St. Louis, and is so well described by Dr. George L. Field, that I will, in the main, use his description. He says that, about six years ago, the patient, a young lady, was brought to his office by her sister to have her right central incisor examined, which was so loose that it could be picked out with the fingers. The doctor said to the young lady that it was broken off directly below the margin of the gum, probably by a severe blow. She denied this, and said that it had been getting loose for a good while. She was a nervous, sensitive young lady, and would not have the tooth removed and a small plate put in. After that she went to St. Louis, and while she was there, the right cuspid, which had never been erupted, presented itself directly through the root of the right central incisor above the margin of the gum. She consulted Dr. Park and Dr. Spalding, in St. Louis, and they advised the removal of the cuspid, and thought there would be enough bone deposited in the socket to make that central incisor fast again. Dr. Spalding wrote me a long letter about it, and the cause of the tooth being loose was thus made apparent: the cuspid ploughing its way through the root, not destroying the pulp, and nutrition going on as before. Drs. Park and Spalding made an effort to extract it, but did not succeed, but broke a small portion from it. After a time they got at the cuspid tooth, but the central incisor still hung loosely. She afterwards consulted Dr. Morrison, of St. Louis, and he said there was a cuspid below which was inside of the arch, on the right side, and he would take out the tooth and bore up into the alveolar process and take out the cuspid below, and put it in the place of the incisor. The young lady was averse to wearing an artificial tooth, and was willing and brave enough to stand any thing, although her temperament was very nervous.

Dr. Morrison bored up through the alveolar process with a large bur and took out the cuspid tooth below, and found the cavity was not large enough, but he made room for the root. After he got the hole large enough and deep enough, he cut off the cusp of the tooth and cut out so that he could fill and give a square form to the edge of the tooth as nearly like a central incisor as possible. The tooth thus prepared was inserted into the socket, and in a short time became about as firmly fixed in place as any tooth in the mouth, and, without a close examination, it would not be distinguished from its neighbor. This tooth, after a year and a half, was as firm in place as any of the incisors, and was not changed in color.

*Dr. H. A. Smith.* In the replantation of teeth, in nearly all cases, the sockets have been diseased, and the subsequent failure of such operations is undoubtedly due to that fact. If the diseased and suppurating tissue of the socket were thoroughly drilled out and a larger tooth put in, we would probably have more success. In Dr. Younger's cases there is no disease of the artificial socket, but at the same time it is not physiological to suppose that the peridental membrane retains life during its dessicated condition. The tooth receives nutrition from the pulp as well as from the peridental membrane, so that is reasonable to suppose that the pulp chamber and canal of an implanted tooth should be sealed up. Absorption of the root is bound sooner or later to occur, and it is yet too soon to say that Dr. Younger's method is a success.

Adjourned.

EVENING SESSION.

*Dr. Jere. Robinson, Sr.* I have transplanted a great many teeth and replanted not more than two or three.

The paper sets forth that the failures in cases of replantation are due to the disease and suppuration previously existing in the socket.

Dr. Younger's method of implanting teeth in an artificial socket has no previous disease to combat; furthermore, the climate of California is more favorable to that class of operations than that elsewhere. My success with replantation has not, on the whole, been satisfactory. I believe that Dr. Younger has established a new principle, and which, in his hands, bids fair to be a success.

*Dr. G. W. Keely.* The first case of replantation I ever saw, was many years ago; the tooth was extracted, cleaned, replanted, and remained healthy for thirty years.

My first case was one of replantation, a lower molar, it lasted fifteen years and finally dropped out; it was my best case.

*Dr. Hall.* I extracted a lower molar, cleaned, and replaced it, but it was extracted by a physician a few days afterwards. In another instance, a superior central incisor remained in for five years, when the root absorbed and the tooth dropped out.

*Dr. Lyder.* In the fall of 1874 I had an alveolar abscess in my own mouth; Dr. Jennings decided the pulp was dead, drilled into the tooth and found the pulp alive. I had the tooth extracted, cleaned, and replaced and it is still in my mouth. Another tooth in my mouth abscessed a year ago, and the pulp in it was also alive. The abscess was situated about half way between the neck and the apex.

*Dr. Harroun* It is possible for an abscess to exist without death of the pulp, though it is rare.

*Dr. D. R. Jennings.* The abscess Dr. Lyder speaks of, was situated about half way between the neck and the apex; the abscess was well developed. Upon extraction of the tooth the pulp was found to be alive and in a very healthy condition. The pulp had nothing to do with the abscess in this case, but if the abscess is on the end of the root at the apex, the nerve will never be found alive.

*Dr. Smith.* Was there a cavity in the tooth?

*Dr. Lyder.* There was a very small gold filling.

*Dr. Smith.* Was there any detachment of the soft parts?

*Dr. Lyder.* Some.

*Dr. Smith.* Why should we have this lesion without some impression or other having been made upon the tooth, such as the irritation incident upon the introduction of a filling or a lesion of the margin of the gum?

*Dr. Jennings.* The margin of the gum around the tooth was firm and hard; pus discharged at a point above from a fistulous opening. There was no sinous opening. If there was or could have been, why should the pus discharge from an opening above the margin of the gum when gravitation would have taken it below?

The tooth was not extracted until a year after the destruction of the pulp and the filling of the canal.

After some further discussion the Society adjourned.

SECOND DAY—MORNING SESSION.

This session was made a special order of business for the demonstration of the Herbst method by Dr. F. H. Rehwinkel, this being the first time the method was shown in practice before this Society. In addition to this many new and novel instruments and appliances were shown by various members.

AFTERNOON SESSION.

This session was also devoted to clinics, Dr. Truman M. Brophy, of Chicago, demonstrating the method of using his matrices for filling approximal cavities in molars and bicuspids. The balance of the afternoon was devoted to the election of officers, a list of which will be found elsewhere in the REGISTER.

EVENING SESSION.

The discussion of Herbst's method was opened with a paper called:

A CRITICISM ON THE HERBST METHOD OF FILLING TEETH, BY DR. W. W. ALLPORT, M.D., D.D.S.

It will be remembered that when Dr. Herbst's method of filling teeth was brought before the American Dental Association a little over two years ago, I then stated, that while not doubting that gold could, under certain conditions, be well packed into a cavity in a tooth by it, I was of the opinion that the objections to the system were so many and important that its general adoption in practice was neither practicable nor desirable. The opinion expressed was not received with much favor at the time, nor is it certain that many agree with me now. I am pleased however to know that Dr. Rehwinkel nearly coincides with me.

Thinking it possible that there might be good reasons for changing this opinion in regard to the matter, I was glad when it was announced that Dr. Herbst was coming to this country to demonstrate his method, and, subsequently that arrangements had been made for his presence at the late meeting of the American Dental Association that we might see him operate and learn from him directly, his reasons for, and the particular advantages of his method.

The acquaintance we made with Dr. Herbst was certainly a most agreeable one. For him, personally, I have the kindest feelings, while for his genius I have great admiration; and my respect for his head and his heart is such, that I do not believe he would desire you nor me to adopt his system of practice, or commend it to others, unless it had the approval of our judgment.

That gold can be well condensed by his system I have not a particle of doubt, nor have I ever had. Neither have I any doubt that most excellent fillings may be made with crystal or plastic gold, for I have put in hundreds and I may say thousands of them. To be frank in regard to this matter I will say, that much of the little early reputation I may have gained as an operator was acquired in the use of crystal gold, for during the first few years of my practice in Chicago I seldom filled a tooth without using more or less of it—this being before the introduction of cohesive foil. But because good operations have been made with it, and can be made with it, is not a sufficient reason for its general use in practice, for experience and observation have taught that quite as good operations can be made with less exactness in details and labor, than is required in the use of this preparation of gold.

With a full acknowledgment of the possibilities that may be attained with this form of gold, the exactness and labor required to reach the highest results in its use is so great, that it was long ago abandoned by a large majority of our best practitioners, though some still make quite extensive use of it, and all might possibly make a limited use of it to advantage. Its general use, however, is neither practicable nor desirable, because quite as good results can be reached with foil, with much less time and labor.

I make this reference to the use of crystal gold for the purpose of illustrating and enforcing my ideas in regard to what is known as the "Herbst method."

Without attempting to discuss or explain the physics involved in the impaction of gold by revolving pluggers (burnishers), I accept the proposition as a demonstrated fact, and will, with your permission, state some of the objections which seem must ever hold against its general adoption in practice.

The first I will mention is the great difficulty and sometimes impossibility of reaching all parts of a cavity with the straight and clumsy instruments that must be used by this method—cavities which could be readily reached and the filling material properly packed with the variously shaped hand or mallet pressure instruments to be found in all well equipped operating cases. In many fissures or deep undercuts that could readily be filled by the old method, a doubt must always be present in the mind of the operator as to whether by this new method such places are perfectly filled or not, for Dr. Herbst himself admits that the only way he can be certain in this matter, is to test the questionable places with small hand plugger points. It requires no argument to show that a plugger which cannot be relied upon to make known a defective place in a filling, cannot with certainty be relied upon to make the filling good at that point, for an instrument which is too clumsy or ill-adapted to find the defect with, is certainly not well adapted to properly fill it when found. If hand or mallet pluggers can be more safely relied upon to find and fill difficultly accessible points about a filling, than can the Herbst method and pluggers, their superiority for filling the balance of the cavity, or for making simple fillings in easily accessible places can hardly be questioned.

Second. To make this method available we are instructed to cut away so much of the sound and strong portions of the tissue as will make all portions of the cavities accessible to these arbitrary and clumsy instruments. To this procedure I object and enter an uncompromising protest, for no one has the right to use an instrument or adopt a system of practice that makes it necessary to sacrifice sound, healthy tooth structure which could be saved by the use of other materials, instruments, or methods of practice.

In preparing a cavity to receive a filling, it is the duty of the dentist to remove all tooth structure that cannot be saved in a healthy and useful condition, but no more. He has the right to cut away as much of the sound structure of a tooth as may be necessary to secure a firm foundation and retention of his filling. But in doing this, it is his duty to see to it that the material used for his filling, is, of its kind, the most easily adapted to the intricacies of the cavity to be filled; also that instruments used in packing it are such as are the best calculated to reach all places in the cavity rendered difficult of access, by virtue of saving portions of the tooth, the removal of which would unnecessarily and forever mar its beauty and harmony. To remove more than this to suit his own convenience, or to enable him to use any particular materials or instruments in filling, might, without a very great stretch of imagination or injustice, be considered malpractice, in a mild form, at least.

While these remarks are made with special reference to the unnecessary sacrifice of tooth substance, by adopting the "Herbst method," I will say parenthetically that they are not without quite too frequent application in regard to fillings put in with cohesive foil. Teeth that could be just as safely filled and much more of the tooth structure saved by a judicious and skillful use of non-cohesive combined with cohesive foil, are in part, mercilessly destroyed in order that the successful use of cohesive foil, exclusively, may be insured.

Admitting therefore, that under certain circumstances, good fillings may be made by the "Herbst method," but to do which in other cases, would require the destruction of more tooth structure than would be necessary in making good fillings by other well known methods, I believe its general adoption in practice is neither desirable nor justifiable.

Third. As "time is often said to be the essence of a contract"—an important factor in the consideration—so too, the time consumed in the use of any particular tools or instruments in doing a given thing when contrasted with the time consumed in producing just as good results with other materials and instruments, is an important factor in wisely choosing the instruments or materials to be used.

In making these remarks, I am aware that the claim is made by some, that much time can be saved by this method; this I do not believe to be true. The same claim was made for crystal gold when it was first introduced, and later, for plastic gold when it came up for our approval. Good fillings, we were told, could with old burs, or most any kind of an instrument, be put in with it, in from two to three minutes. In neither case, however, has experience proven the claim to have been well founded; on the contrary more time is consumed in putting in good fillings with either of these materials than is required with foil. The same history is to repeat itself in regard to the "Herbst method." No doubt fillings can be *wiped* in by this process very quickly, but the highest results from it will only be reached after much painstaking labor.

If, therefore, as I believe is the fact, under favorable circumstances more time is consumed in making good operations by the "Herbst method" than by those just mentioned, the method is neither practicable nor desirable in this practical and time-saving age, for time is money, not only to the dentist but also to the patient.

Fourth. That quite as good, or even better operations can be made under circumstances favorable for the "Herbst method," by the use of suitable instruments and non-cohesive foil enameled with cohesive foil, under mallet pressure, and without the necessity of sacrificing so much sound tooth structure.

Personally I have had but little experience with these rotary pluggers, but I have seen Dr. Herbst and my friend, Dr. Rehwinkel operate, and I have witnessed some of Dr. Marshall's experimental operations by this method, and I confidently assert that I have seen better fillings made by hand mallet and the old fashioned hand pressure methods, and that too, in much less time than was consumed in making the fillings by the " Herbst method." My observation has thus far led me to the belief that there is no saving of time or superiority of operation to be obtained by the exclusive use of the new method over the old systems. Therefore, as a matter of economy, or superiority of results, I see nothing to justify its general adoption.

While the objections to this method as a general system of filling teeth, are so obvious that its adoption is not warranted, it is possible that as an adjunct to other methods of practice it may be made useful ; in fact, I believe it can.

From what I have seen, I think it can be made useful at the cervical walls of cavities, especially when the matrix is used. The surface of the pluggers being smooth, and the impact made without violent concussion, when proper access can be had, it could be advantageously used in packing gold against margins of cavities, preventing the fracturing of the enamel which so frequently occurs in the use of the mallet pluggers. No doubt it may be advantageously used in other places, but those mentioned are where its greatest excellence will be found. Could a wise judgment be exercised in determining when and where it could, or could not be used with benefit, I have no doubt that it might with advantage be occasionally employed. The fear, however, is that an over zealous advocacy of the system by its friends will lead many to use it when better results could be secured by the old methods. By its general adoption, or too frequent use, frequent failure would be the result, and the method brought into undeserved disrepute, with its abandonment, even while it could be used to advantage, thereby losing, in a measure, at least, the benefits that might follow from a judicious advocacy and use of it.

Now, Mr. President, having stated to you freely and frankly some of my objections to this method of practice, I trust no one will think I have had the least intention of being unkind or harsh to any one, for nothing could be farther from my desire. I

have only intended by words of truth and soberness to make my views clear to you—to be approved or disapproved by argument, or the test of time, as truth may justify.

For Dr. Herbst, I have a great admiration. He is more than a Yankee in invention—as generous as King Lear in giving; nor is he so conceited that he cannot learn from others; an enthusiast but in no sense an egotist, he freely gives and is wise enough to freely receive. As a profession we are under many obligations to him for the many useful inventions and "tricks" if you please, that he has left with us, and we shall gladly welcome his promised return to this country next year.

*Dr. Rehwinkel.* Dr. Allport is doubly particular to state that no time can be saved in filling teeth by the "Herbst method;" I cannot accept that. Dr. Allport did not see Dr. Herbst operate at his best as there were very many present, and he was continually interrupted to explain this, that, and the other step in the process. At Dr. Boedecker's office, where there were fewer present, and notwithstanding the many questions asked him, and the time consumed in translating his remarks into English, the whole time occupied in the filling was but ten minutes, while the same work would occupy Dr. Allport at least twenty-five minutes. You must not judge of the time required by the operation I made to-day, as I consumed a great deal of time answering questions and showing and explaining the various steps as I went along.

Dr. Herbst can fill the smallest cavity in a lower incisor, and with the least operating space, so that this fact will refute Dr. Allport's statement that Herbst's instruments are clumsy and ill-adapted. The instruments he uses are made in all sizes, some of them as fine as needle points. Dr. Herbst adjusts the matrix *before* he commences to excavate the cavity, as in that way, he preserves the shape of the tooth and a great deal of tooth-structure, though we are divided in opinion as to how much should be sacrificed or saved. Dr. Allport must have been mistaken or misinformed. I agree with him, however, that few in this country will become as enthusiastic as Dr. Boedecker.

A great advantage of Herbst's system is doing away with retaining pits, thus preventing the crushing and breaking down of the cervical border.

The gold can be thoroughly adapted into the most minute crevices and irregularities of a cavity, and more completely than by any other method. No one has learned to fill by any method in a day or two, but devoted much time and trouble to acquire the necessary proficiency, so too must the "Herbst method" be thoroughly mastered by continued practice.

There is something in this method worth looking at, whether you adopt it as a whole or in part; it is a genuine acquisition to operative dentistry.

*Dr. J. Taft.* I can vouch for what Dr. Rehwinkel says. I witnessed Dr. Herbst's operations with a great deal of interest, and I confess I went there rather prejudiced against the method; this was removed, however, upon seeing the man, his manner, freedom, etc. The impression with many is that he uses rotary instruments only; this is not true, as he also uses hand instruments, and is very careful in examining the borders as he goes along, with a sharp pointed instrument. In all inaccessible localities of the cavity the filling was perfected with hand instruments. Dr. Rehwinkel mentioned that Dr. Herbst lined or varnished the inside of a cavity with gold before introducing the filling proper. I can see no objection to that. The thorough adaptation by this method must certainly hermetically seal the cavity. The liability of comminuting or fracturing the borders of a cavity is certainly less than with the ordinary plugger, as there is no sudden impulse given to the frail wall of the cavity, consisting of enamel only. The rapidity of execution is certainly greater than by malleting in pieces one at a time. There are undoubtedly advantages in the system that we might adopt with profit.

*Dr. J. R. Callahan.* The gentlemen who have spoken upon the subject have not made clear the use of the cotton placed over the gold. Does it rotate with the instrument?

*Dr. J. Taft.* No, it remains steady, the instrument passing over it.

*Dr. Butler.* I have not seen mentioned by either Herbst, Bodecker, Taft, or Rehwinkel, the principle of having a continuous wall. If it does not exist in the tooth, it must be made by the introduction of a matrix. None have mentioned what would become of the mass of gold if a portion of the wall should give way, the cavity being saucer shaped, or on a plane surface like the bottom of this glass. Angles or grooves should undoubtedly be made to retain the mass in case of fracture of a wall, and to sustain the weight and tear of mastication.

The fact that gold can be carried into the cuts and grooves of a medalion is not new. The spinning of metals is a very common thing amongst artisans. In a practical sense, it is questionable whether the filling is a good one without angles and corners to retain its position and integrity. I doubt if a stiff matrix will allow the making of as good borders as a matrix that will yield a little, and right there is where Herbst secures an advantage in using copper and German silver. There is not as much tooth substance sacrificed by this method as by those who use the electric mallet, in getting as they must, direct and thorough access to the cavity.

*Dr. Truman W. Brophy.* The use of soft gold, or I may say its reinstatement, will result in great good to the profession, as we are thereby enabled to perform more and better operations than with cohesive foil and the mallet. The drilling of the retaining pit (improperly so called) causes the patient more pain than all the rest of the preparation of the cavity. No retaining pits, grooves, or angles are necessary, and I don't need a square base as was formerly insisted upon, in fact I don't care how the cavity is shaped. The principle objection I had to Herbst's method was his matrix; if the matrix is unyielding you cannot cover the borders with the gold; you must have a yielding matrix and carry the gold over the border between the band and the tooth with a wedge-shaped plugger, such as was designed by Dr. Black.

*Dr. J. Taft.* What was the object Dr. Brophy, of using the tin and gold combined, as you did?

*Dr. Brophy.* Dr. Miller, of Berlin, investigated the method in a scientific manner and claims that the adaptation is easier and more perfect than when gold alone is used, and in addition, the combination exerts some peculiar prophylactic influence; it also becomes harder than gold, after a time, in fact crystalizes.

*Dr. Allport.* I am aware that some of the instruments that Dr. Herbst uses are small; I referred more especially to their inadaptibility in inaccessible corners and angles inasmuch as they are straight.

*Dr. Rehwinkel.* Dr. Herbst uses a right-angle hand-piece to reach such places as Dr. Allport mentions.

*Dr. Allport.* Quite likely, Mr. President, I had better not have made use of the word clumsy, which Dr. Rehwinkel takes exceptian to, when I referred to Dr. Herbst's instruments, for, as he says, many of them are very small. Illy adapted, I presume would have been a better term, for straight instruments, no matter how small or delicate they may be are not well adapted to working around corners, or into deep or hidden under-cuts, any more than the straight course of a ball from a rifle is calculated to follow the angles or crooks in a rail fence.

As reference has been made here to the practice of combining gold and tin foil as a filling material by folding two or more leaves together because they can be more easily packed than gold alone, and also that in time the two metals become crystalized and hard, I want to say that while both of these claims are true, they are, in my mind, not the most important reasons for the practice. It is said that it makes no difference which of the two metals is folded upon the outside and comes in contact with the tooth. This I believe to be a mistake, for the sulphide of tin produced by the combined action of these metals, and the fluids of the mouth, is a powerful antiseptic. The product being insoluble in the fluids of the mouth, its action is not only continuous in preventing decomposition, by virtue of its antiseptic properties, but it also acts as a permanent filling, and makes good, or fills up minute defects between the filling and the tooth—an advantage not so fully derived when the gold is placed in contact with the tooth. Gold alone has no beneficial therapeutical effect upon a tooth, while tin has. Tin not only prevents decay by preventing the ingress of the fluids of the mouth, but by its antiseptic properties. In using these metals in combination therefore, I always place the tin upon the outside, or next to the tooth. Forty years of experience and observation teaches me that, largely, by virtue of its chemical products in the mouth, of all the filling material known, none acts more kindly upon the dentine, or is so sure to prevent or arrest decay, as tin.

*Dr. Harlan.* This subject is of great interest, but the principle of planishing gold into cavities is not new by any means, inasmuch as Shumway and Chance both have advocated the use of planishing points many years ago. Why, Prof. Taft here, at a meeting of the Illinois State Dental Society, at Jacksonville, in 1874, was announced to fill a tooth with heavy foil, planishing the gold into place piece by piece and he did it. Am I not right Dr. Taft?

*Dr. J. Taft.* Yes.

*Dr. A. W. Harlan.* Dr. Allport says that Herbst's method involves the sacrifice of a large amount of tooth-structure, that may be, so did the old method of making fillings with non-cohesive gold involve the cutting away of immense amounts of tooth substance; hence, this is no argument against its adoption. Any method of filling necessitates the cutting away of sound tooth substance to a greater or less extent. In my estimation, nothing can supplant hand pressure and the mallet.

Any method requiring a band or matrix to hold the filling in place during the process of its introduction cannot commend itself to the plain, common-sense, every-day-working dentist. It is the most misleading doctrine that has ever been propounded to students and the younger and inexperienced practitioners, inducing as it does the most slovenly methods. Herbst's method cannot serve to fill one half of the cavities we are called upon to fill, and this is especially true of contour operations.

If you take four or eight sheets of non-cohesive gold and subject them to great pressure, you will find after its removal that the sheets can be separated, showing that this is true. Herbst's method is an excellent thing for German dentists, as many of them do not use one-eighth of an ounce of gold in a whole year.

There is no doubt but that it will do away with the black amalgam fillings one sees in the front teeth of native Germans.

I have the greatest admiration for Herbst, his ingenuity, and generosity, and regret that his method cannot command my full support. Gold which is non-cohesive, cannot be depended upon for mastication, as the element of strength is lacking in all contour fillings. It is absolute folly to adopt a method with which we are unacquainted and discard old and tried systems which fulfill every indication. Herbst and his followers confess that it is better to finish the filling with cohesive gold and the mallet.

If it is so practical, any good operator can fill a tooth by the present methods as quickly as by this new method.

Tin is not so good a conductor as gold, and that is one reason why it should be used.

In reference to the formation of sulphide of tin, Dr. Allport is perfectly correct. Tin and gold combined, harden very materially after a time.

*Dr. Dorrence.* The use of tin is a most excellent procedure, not only because it lessens the shock from thermal changes, but because it also hardens the dentine.

*Dr. Land.* During the past four years, I have found upwards of twenty cases of crystalization of the combination, and furthermore, the tin is the base of an antiseptic.

*Dr. G. W. Keely.* I fully convinced myself of this twenty years ago.

*Dr. Brophy.* I do not think it is fair to say that Dr. Herbst relies upon the matrix to hold the filling in place while it is being introduced and condensed, it is more to give shape to the filling.

Adjourned.

THIRD DAY—MORNING SESSION.

*Dr. Jere. Robinson*, of Jackson, Michigan, read a paper on "Operative Dentistry, (which will appear in the next number—Ed.)

*Dr. Siddall.* Undoubtedly the first requisite is to *see* clearly what you are doing, and for men at my time of life, it is necessary to have proper glasses, nicely adjusted to the sight; my first pair of glasses came in the nature of a revelation to me, enabling me to see many little things that had long escaped me. The eye-glass, such as jewellers use, is also of valuable assistance to the operator.

*Dr. Butler.* A great deal has been said here concerning the good qualities of tin, and I am glad to see there is so much harmony on the subject. Regarding the use of textile foil by the profession, I asked Dr. Robinson whether the demand for it was increasing, and he told me that it conflicted too much with amalgam, in the hands of the dealers, to increase in use to any considerable extent. I opine the trouble is more with the operator than the material.

For myself, I have been using it ever since its introduction, up to the day I left home; it is one of the things you like better the more you use it, and I hope it will come into general favor. When thoroughly packed, it makes a mass nearly as solid as cohesive foil.

*Dr. Harroun.* It must be packed very thoroughly and burnished afterwards.

*Dr. Robinson, Sr.* I had a long controversy with Prof. Flagg, who said, after analysing it chemically, that there is no gold in it. I sent him another specimen for analysis and he found the gold in it. I pledge you my word, gentlemen, that it is composed only of tin, gold, and platinum.

*Dr. Dorrance.* A little point I mentioned last night was not "caught onto" as the phrase is. Instead of using a silk ligature to carry the rubber dam beyond the margin of the gum, use a little binding wire, pass it around the tooth, twist it tight, and you will thus be able to carry the rubber away beyond the cervical border under the free margin of the gum.

*Dr. Callahan.* I have used Dr. Robinson's textile foil for five or six years, and I now find I can make it much more solid by the rotary plan of Dr. Herbst, than I can with the mallet.

*Dr. Smith.* I believe Dr. Harlan said something, or at least mentioned Dr. Herbst's obtundent, and I should like to hear him more fully on the subject.

*Dr. Harlan.* Herbst's formula for his obtundent, as nearly as I can recollect, is as follows:

℞ Cocaine hydrochlorate,........................grs. xxx.

Acid Sulphuric....................................ʒ ii.

Spts. Ether Sulph.................................ʒ ii. ss.

From experiments made in Chicago, it was found that more than seventy grains of cocaine were required to saturate two drachms of sulphuric acid. It was also found that a tooth immersed in pure sulphuric acid for a week showed no effects of the acid upon the enamal, so that no fears need be entertained that the acid of the obtundent will do the tooth any damage. It was further learned that repeated applications were necessary, as it is self-limiting, penetrating the superficial layer only. It was discovered also that ten grains of the alkaloid (cocaine) dissolved in 90 ♏ sulphuric ether (the acid left out) would, in one application, act quickly and more thoroughly; so it appears from this, that it is better to leave the acid out altogether. Fifteen or twenty grains of the alkaloid may be used if desired. Ether is a refrigerant, evaporates rapidly, and is the most useful solvent, as the alkaloid cocaine is soluble in it in all proportions.

*Dr. Smith.* Is the action on the fibrils of the dentine or on the nerve distribution directly to the pulp?

*Dr. Harlan.* I don't know.

*Dr. Smith.* Our treatment of super-sensitive dentine appears to be based upon the supposition that dentine contains nerves. They have not been demonstrated. If the dentinal fibrils are nerves, we should be able to get more uniformly the results obtained in local use of sedatives upon tissues known to contain nerve distribution. If regarded as processes of the odontoblasts and protoplasmic in their nature, as taught by Prof. Black and others, some of the phenomena observed in the use of obtundents may be explained.

Protoplasm possesses contractility, and therefore is sensitive. This contractility is readily influenced by changes in temperature, by the abstraction of moisture, by acid and alkaline reagents; also by chloroform and ether. We try all of these in turn, and fail in controlling sensitiveness, because of the peculiar structure of dentine. The action of arsenious acid on dentine is mysterious. When applied even superficially on dentine it, as a rule, soon destroys the vitality of the tooth. The rapidity of its action appears to show that arsenic is transported directly to the highly vitalized tissue, the pulp. May not this be accomplished by the phenomenon of *intussuseption?* The dentine contains protoplasm—the dentinal fibrils. When any minute foreign particles come in contact with protoplasmic substance they first adhere and then are drawn into the interior and carried on by the currents which exist in the protoplasmic substance. The arsenious acid in minute form is brought in contact with protoplasm—the fibrils, and quickly transported to the pulp, where it manifests its usual toxic effects.

*Dr. Butler.* Do you think it possible for arsenic to be drawn into the tubules if it is applied in the dry state?

*Dr. Smith.* If my theory is correct, the dry powder is best.

*Dr. Butler.* The old theory is, that a medicament must be absorbed before any effect can be produced.

*Dr. J. Taft.* Chloride of Zinc was formerly used as an obtundent, in some instances it allayed the pain of excavation, in others it seemed to enhance it. In some cases the obtunding was perfect, in that the tissue dies, while in others the pain returns. There are other agents that may be employed with effectiveness, and which do not destroy or change the tissue, for instance, oil of cloves will sometimes prove efficient.

It is true, as Dr. Harlan stated, that sulphuric acid will not act upon a tooth immersed in it; if, however, the acid should be diluted, or heat applied, roughening will take place. Sulphuric acid is an escharotic.

The obtunding power of Dr. Herbst's preparation is excellent in some cases, *nil* in others, just as is true of all our obtundents." Subject passed.

*Dr. Talbot.* I wish to call your attention to a method of regulating teeth by means of piano wire springs adjusted to a couple of small plates of vulcanite fitted against the teeth to be moved. The young lady here is a patient of Dr. Harroun's, and I made the plates upon a model he sent me so that it could be exhibited to you. The little plates each have a hole drilled into them, into which fits a nib at the end of the spring so that it can swing forwards or backwards to suit the convenience of the patient. Instead of the W spring, the wire is coiled upon itself two or three turns, in the middle, and then bent into the form of a V; the nib at each arm of the V fitting into the holes; the coil will be at the junction of the two arms. The plates are, of course, to be securely tied into place. Until four years ago, the lever, screw, wedge, inclined plane, and wheel and axle, were the mechanical forces used in regulating teeth. Piano-wire in the form of springs of various shapes has since come into great favor, the curves and coils into which it is formed giving the necessary force; the pressure is constant, and one spring will answer for many cases. In spreading an arch, for instance, it requires some little study to determine where to drill the holes in the rubber plates I spoke of. If the holes are deep enough, the force of the spring will be sufficient to retain it in place, though the plates must be securely ligated to the teeth.

*Dr. Geo. W. Keely* exhibited many models and paintings of simple and complicated cases of irregularities he has treated from time to time, giving a full explanation of the plan pursued in each individual case. His remarks being altogether descriptive, and cases following each other in rapid succession, it was simply impossible for the reporter to do justice to the subject.

The afternoon was consumed in transaction of miscellaneous business, and the Society adjourned, to meet again last Tuesday of October, 1887, at Springfield.

